# Genetic Diversity and Primary Drug Resistance of *Mycobacterium tuberculosis* Beijing Genotype Strains in Northwestern Russia

**DOI:** 10.3390/microorganisms11020255

**Published:** 2023-01-19

**Authors:** Anna Vyazovaya, Alena Gerasimova, Regina Mudarisova, Daria Terentieva, Natalia Solovieva, Viacheslav Zhuravlev, Igor Mokrousov

**Affiliations:** 1 Laboratory of Molecular Epidemiology and Evolutionary Genetics, St. Petersburg Pasteur Institute, 197101 St. Petersburg, Russia; 2 St. Petersburg Research Institute of Phthisiopulmonology, 191036 St. Petersburg, Russia; 3 Henan International Joint Laboratory of Children’s Infectious Diseases, Children’s Hospital Affiliated to Zhengzhou University, Zhengzhou 450018, China

**Keywords:** *Mycobacterium tuberculosis*, primary multidrug resistance, Beijing genotype, Central-Asian/Russian clade, B0/W148

## Abstract

The Beijing genotype is the main family of *Mycobacterium tuberculosis* in Russia. We analyzed its diversity and drug resistance in provinces across Northwestern Russia to identify the epidemiologically relevant Beijing strains. The study collection included 497 isolates from newly-diagnosed tuberculosis (TB) patients. Bacterial isolates were subjected to drug-susceptibility testing and genotyping. The Beijing genotype was detected in 57.5% (286/497); 50% of the Beijing strains were multidrug-resistant (MDR). Central Asian/Russian and B0/W148 groups included 176 and 77 isolates, respectively. MDR was more frequent among B0/W148 strains compared to Central Asian/Russian strains (85.7% vs. 40.3%, *p* < 0.0001). Typing of 24 minisatellite loci of Beijing strains revealed 82 profiles; 230 isolates were in 23 clusters. The largest Central Asian/Russian types were 94-32 (*n* = 75), 1065-32 (*n* = 17), and 95-32 (*n* = 12). B0/W148 types were 100-32 (*n* = 59) and 4737-32 (*n* = 5). MDR was more frequent in types 1065-32 (88.2%), 100-32 (83.1%), and 4737-32 (100%). In contrast, type 9391-32 (*n* = 9) included only drug-susceptible strains. To conclude, *M. tuberculosis* Beijing genotype is dominant in Northwestern Russia, and an active transmission of overwhelmingly MDR B0/W148 types explains the reported increase of MDR-TB. The presence of MDR-associated minor variants (type 1071-32/ancient Beijing and Central Asia Outbreak strain) in some of the studied provinces also requires attention.

## 1. Introduction

*Mycobacterium tuberculosis* has a clonal population structure that includes nine phylogenetic lineages, of which Lineage 2 (or East-Asian lineage) is one of the most widespread. The Beijing genotype (L2.2) is a main component of Lineage 2. It is presently found on all continents but is most common in Asia and Europe [1,2,3]. At the same time, in countries with a high burden of tuberculosis (TB), Beijing strains are associated with drug resistance, including primary multidrug resistance (MDR), i.e., resistance to at least rifampin and isoniazid [3]. A higher adaptive capacity and resistance to anti-TB drugs in Beijing strains compared to other genotypes were attributed in some studies to a high mutation rate in vitro and in vivo although this issue remains controversial [4,5,6]. The Beijing genotype is divided into phylogenetic sublineages (ancient/ancestral and modern), based on variation associated with some genome regions, such as IS*1547*, *Rv3135*, RD181, NTF, and SNPs in *mutT2* and *mutT4* [7,8,9,10,11,12].

The *M. tuberculosis* Beijing genotype is associated with insufficient treatment efficacy and recurrence of the disease due to an increased ability of these strains to acquire drug resistance, although this is variable across Beijing subtypes and countries [13,14,15,16]. Similarly, different Beijing strains vary in their virulence properties. Beijing strains have an increased ability to inhibit protective immunity in the lungs by inducing higher levels of type I interferons, which lead to a decrease in the level of some pro-inflammatory cytokines, reducing T-cell activation [13]. However, single Beijing isolates do not represent all the diversity of the Beijing genotype, and even within the same group of related isolates a diversity of their virulence was shown in murine model studies [16,17].

The differentiation of sublineages and clusters of the Beijing genotype is not only a matter of classification but is of clinical and epidemiological significance. In Vietnam, resistance to isoniazid and streptomycin was more often observed in Beijing strains of the ancient sublineage [14]. In Russia, two MDR/pre-extensively drug-resistant (pre-XDR) clusters of ancient Beijing sublineage have recently been identified in Siberia and Russia Far-East within RD181-intact (Multi-locus VNTR analysis [MLVA] type 14717-15) and RD181-deleted (type 1071-32) groups [15]; the former was hypervirulent and highly lethal in a murine model [16]. Among predominant modern strains of the Beijing genotype in Russia, two large clusters are the most known: the notorious epidemic strain B0/W148 (~CC2 clonal complex [2], type 100-32) and Central Asian/Russian clade (~CC1 [2], type 94-32). Their 24 mycobacterial interspersed repetitive units-variable number of tandem repeats (MIRU-VNTR) profiles differ in MIRU26 and QUB26 loci (7 and 7 repeats for type 100-32; 5 and 8 repeats for type 94-32). An important strain within the Central Asian/Russian clade is the so-called Central Asia Outbreak (CAO) strain, which is MDR and endemically prevalent in Central Asia, but as of yet appears infrequently in European Russia [18]. Whereas the epidemiological and clinical significance of the B0/W148 cluster in Russia was demonstrated by numerous studies, the role of the more common and heterogeneous Central Asian/Russian clade and its CAO subtype remains to be assessed.

Here, we performed the molecular characterization of *M. tuberculosis* isolates of the Beijing genotype and its major endemic and epidemic subtypes in different regions across Northwestern Russia. We analyzed isolates recovered from newly diagnosed patients to understand what strains are actively transmitted and therefore impact the current situation of MDR-TB in Russia and, potentially, in neighboring countries and countries that receive Russian migrants and tourists.

## 2. Materials and Methods

### 2.1. Study Setting and Bacterial Strains

The survey area included six provinces of Northwestern Russia (https://en.wikipedia.org/wiki/Northwestern_Federal_District, accessed on 10 January 2023). Most of the studied regions have a border with the European Union. The Republic of Karelia has a long border with Finland. The Kaliningrad region in the westernmost area of Russia (semi-exclave) borders Poland and Lithuania. Most of the Murmansk region is located north of the Arctic Circle on the Kola Peninsula and has a common border with Finland and Norway. Finally, the Pskov region borders the EU (Estonia and Latvia), as well as Belarus.

The main criteria for inclusion of *M. tuberculosis* isolates/TB patients in the study were: (i) newly diagnosed patients with pulmonary TB; (ii) culture isolation between January 2014 and December 2018 (for most regions it was a one-year period); (iii) first available *M. tuberculosis* culture isolated prior to treatment; (iv) patient age ≥ 18 years old; (v) availability of accompanying information (gender, age, diagnosis).

A total of 497 strains of *M. tuberculosis* were studied from six regions of Northwestern Russia: Karelia (*n* = 67, 2014–2015), Kaliningrad (*n* = 73, 2015), Murmansk (*n* = 67, 2017), Komi (*n* = 130, 2017), Pskov (*n* = 78, 2018), and Vologda (*n* = 82, 2018). These isolates included all available samples within the survey period and corresponded to the above criteria of sampling.

### 2.2. Drug Susceptibility Testing

*M. tuberculosis* drug susceptibility testing for first- and second-line drugs was performed using a modified proportion method on Middlebrook 7H9 liquid culture and Bactec MGIT 960 system (Becton Dickinson, Sparks, MD, USA) according to the manufacturer’s instructions. The critical drug concentrations used were 1.0 μg/mL for streptomycin, 0.1 μg/mL for isoniazid, 5.0 μg/mL for ethambutol, 1.0 μg/mL for rifampicin, 100 μg/mL for pyrazinamide, 1.0 μg/mL for amikacin, 2.5 μg/mL for capreomycin, 2.0 μg/mL for ofloxacin, and 5 μg/mL for ethionamide.

### 2.3. Genotyping

*M. tuberculosis* DNA was extracted as described previously according to the standard protocol [19]. Initially, the strains were differentiated into Beijing and non-Beijing strains based on PCR detection of the Beijing genotype-specific IS*6110* insertion in *dnaA*-*dnaN* regions as described [20]. The main Russian Beijing subtypes (B0/W148-cluster, Central Asian/Russian cluster, and CAO strain) were detected by PCR-RFLP or real-time PCR analysis of the specific genome regions as described previously [18,21,22,23].

Beijing sublineages (ancient, early ancient, modern) were detected by PCR/gel-electrophoresis analysis of the RD181 deletion and NTF locus (presence/absence of IS6110) as described [10,12,24].

Typing of 24 minisatellite MIRU-VNTR loci was performed according to Supply et al. [25]. The obtained profiles were assigned MLVA codes through comparison with MIRU-VNTRplus.org online tool. In addition, the profiles were assigned to the clonal complexes (CC) within the Beijing genotype through comparison with the large dataset of the Beijing genotype [2]. Of note, some of the VNTR-based CCs described in the study of Merker et al. [2] are polyphyletic and unsupported by robust whole-genome sequencing (WGS) data, e.g., CC3 and CC4.

Hunter Gaston Index (HGI) was calculated at http://insilico.ehu.es/mini_tools/discriminatory_power/index.php (accessed on 10 December 2022). The Shannon index was calculated at https://www.easycalculation.com/statistics/shannon-wiener-diversity.php (accessed on 10 December 2022). The two indices were calculated since more widely used HGI places more weight on species evenness than it does on richness. On the other hand, the Shannon index quantifies the uncertainty in predicting the species identity of an individual that is taken at random from the dataset and accounts for both the abundance and evenness of the species present [26].

The level of clustering (clustering rate) of strains was estimated by the formula: CR = (*n*_c_ − c)/*n*, where *n*_c_ is the total number of clustered strains, c is the number of clusters, and *n* is the total number of strains [27]. In this study, a MIRU-VNTR cluster was considered a group consisting of two or more strains identical in 24 loci. The clustering rate served to assess the diversity of the total and regional collections based on all tested VNTR loci.

## 3. Results

### 3.1. Main Genotype Clusters

The 497 *M. tuberculosis* isolates were recovered from 497 pulmonary TB patients. The main diagnoses were infiltrative (66.2%) and disseminated (18.8%) TB followed by fibrous-cavernous, focal, and other TB forms (caseous pneumonia, etc.) together were diagnosed in 15%. Three hundred and fifty-three patients were male (71%, 18 to 88 years old, mean age 43.7 years), and 144 were female (29%, 18 to 86 years, mean age 43.0 years).

The Beijing genotype was identified in 286 (57.5%) of 497 *M. tuberculosis* strains. Eight isolates were assigned to the ancient Beijing sublineage (intact NTF) while other Beijing isolates belonged to the modern Beijing sublineage (NTF::IS*6110* allele). In the Beijing structure, the most numerous genetic groups were Central Asian/Russian, including the CAO subtype (61.5%; 176/286 strains) and B0/W148 (26.9%; 77/286). The proportion of the CAO subtype of the Central Asian/Russian clade was 10.8% (19/176), or 6.6% of all Beijing isolates. The six regions differed significantly (*p* < 0.001) in the number of *M. tuberculosis* strains of the Central Asian/Russian and B0/W148 groups (Table 1).

The largest proportion of the Central Asian/Russian clade (66.7%), as well as the smallest proportion of the B0/W148 cluster (11.8%), were found in the Beijing collection of Vologda. In Karelia, Komi, and Kaliningrad, the proportions of B0/W148 cluster strains exceeded one-third of the entire Beijing population.

### 3.2. High-Resolution Population Structure

The heterogeneity of the studied Beijing subtypes was assessed by 24-loci MIRU-VNTR typing. Table 2 presents HGI values for each of the 24 MIRU-VNTR loci of Beijing Central Asian/Russian and B0/W148 subtypes. In Central Asian/Russian strains (*n* = 176), the QUB26 (HGI 0.382) and ETRA (HGI 0.224) loci were the most variable; 9 (37.5%) of 24 loci were homogeneous (HGI 0). Strains of B0/W148 (*n* = 77) represented a more homogeneous group compared to Central Asian/Russian strains, since they did not differ in 15 (62.5%) of the 24 MIRU-VNTR loci. MIRU31 was the most polymorphic locus in B0/W148 strains (HGI 0.123), although its value was very low.

The 24-MIRU-VNTR diversity assessed with HGI was 0.713 for all Beijing strains. For particular genotypes, it differed significantly, and was 0.83 for the Central Asian/Russian group (with and without CAO strains), compared to the very low diversity of the CAO group (0.47) and B0/W148 (0.41).

**Figure 1:**
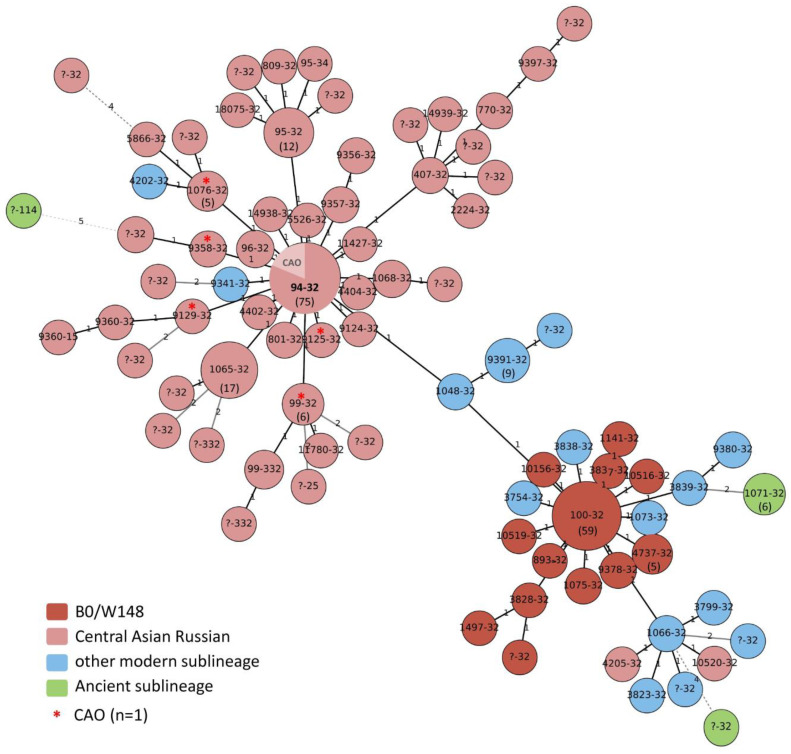
Minimum spanning tree of 24-MIRU-VNTR profiles of 286 strains of *M. tuberculosis* Beijing genotype strains. Node sizes only roughly correlate with the sample size. The number of isolates in the largest types (*n* > 4) is shown inside the circles in brackets. Designations of MIRU-VNTR profiles are given according to the MLVA Mtbc15-9 nomenclature. Lines between nodes show changes related to 1 locus variation (solid bold lines), 2 loci variation (solid thin lines), and >2 loci variation (dashed lines). Numbers on the lines show the number of variant alleles.

24-MIRU-VNTR typing of 286 Beijing strains revealed 82 variants of digital profiles (Supporting Table S1). Of these, 24 profiles were represented by clusters, including from 2 to 75 strains, which amounted to 80.4% (230/286) of strains. The remaining strains each had individual profiles. The minimum spanning tree of the VNTR profiles is shown in Figure 1.

Twenty-five isolates of the modern Beijing sublineage did not belong to Central Asian/Russian or to B0/W148 groups and were grouped into 4 clusters: 9391-32 (9 strains), while 9380-32, 1066-32, and 1048-32 included 2 strains each and 10 individual profiles.

Six isolates of the ancient Beijing sublineage were assigned to type 1071-32, and two other ancient Beijing isolates had unique 24-MIRU-VNTR profiles. Previously, it was shown that 1071-32 strains belong to the early ancient sublineage of the Beijing genotype characterized by *mutT2*-58 and *mutT4*-48 wild-type alleles and deleted RD181 region [15].

Among 82 MIRU-VNTR24 profiles of 288 Beijing strains, 59 types were identified according to the MIRU-VNTRplus nomenclature. The 24-MIRU-VNTR profiles of Central Asian/Russian and B0/W148 subtypes are shown in Table 3.

Most of the B0/W148 strains (68 out of 77) belonged to four MIRU-VNTR clusters, the largest being 100-32 (59 isolates). A total of 52 MIRU-VNTR profiles were identified in 176 Beijing Central Asian/Russian strains: 37 isolates had individual profiles and 139 were in 15 clusters. Of these, the three largest types (*n* > 10) were 94-32 (*n* = 75), 1065-32 (*n* = 17), and 95-32 (*n* = 12) (Table 3). The majority (15 of 19) of CAO isolates had a 94-32 profile, which is a basal profile of the Central Asian/Russian clade. The other 4 isolates were single locus variants of type 94-32. In this view, 24-loci typing was not suitable to distinguish CAO isolates from other Central Asian/Russian isolates.

**Figure 2:**
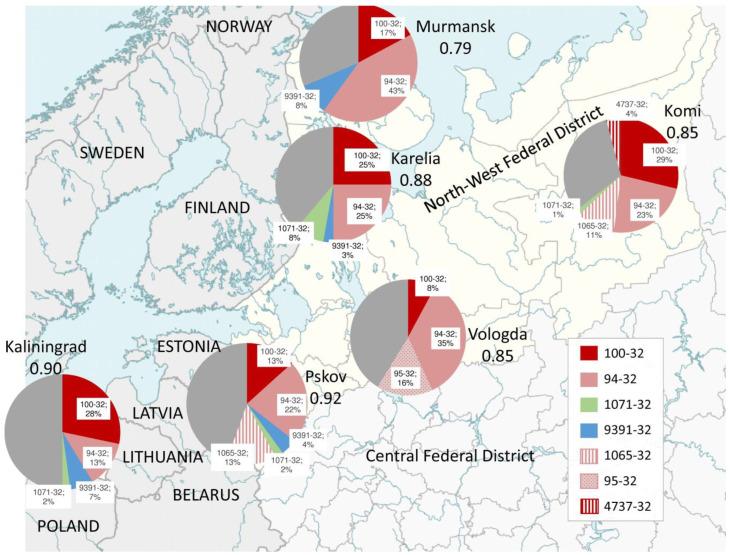
Geographic distribution of the Beijing VNTR types in six provinces in Northwestern Russia. HGI values are shown below the regions’ names. Free map: https://commons.wikimedia.org/wiki/File:Outline_Map_of_Northwestern_Federal_District.svg (accessed on 1 December 2022).

The largest MIRU-VNTR clusters of *M. tuberculosis* Beijing genotype were analyzed in the regional subpopulations (Figure 2; Table 4). The B0/W148 type 100-32 accounted for 25–28% of strains in Kaliningrad, Komi and Karelia compared to only 7.8% of strains in Vologda. In Komi, B0/W148 type 4737-32 (4.1%) was also identified in a minor proportion. Central Asian/Russian type 94-32 predominated in Murmansk (42.9%) and Vologda (35.3%) as opposed to making up 13.0% in Kaliningrad. Central Asian/Russian strains were also represented by cluster 1065-32 in Komi, Pskov, and Vologda (where cluster 95-32-16% was also found). CAO isolates were found in relatively high proportion in Vologda (13.7% of all Beijing), Kaliningrad (8.7%), and Karelia (8.4%) compared to only making up only 2–4% of isolates in Komi, Murmansk, and Pskov. Regarding Beijing isolates beyond B0/W148 and Central Asian/Russian, type 9391-32 of the modern Beijing sublineage was present in Murmansk, Kaliningrad, Pskov, and Karelia. Strains of type 1071-32 of the ancient Beijing sublineage were found in 8.3% of the Beijing population in Karelia and were very low prevalent or absent in other regions.

The VNTR diversity of regional populations is also summarized in Table 4 in terms of the number of different profiles and clusters and assessed using Hunter Gaston and Shannon indices. Both indices (reflecting type richness and evenness), as well as the CR index, concordantly indicated the lowest diversity of the Murmansk collection and the highest for Pskov and Kaliningrad.

### 3.3. Drug Resistance versus Genotypes

The Beijing subtypes differed in phenotypic drug susceptibility (Table 2). The vast majority of drug-susceptible Beijing strains were represented by strains of the Central Asian/Russian cluster (97.1%), while no susceptible B0/W148 strains were found. The Central-Asian/Russian CAO subtype strains were all drug-resistant, and 57.9% were MDR (Table 5).

The largest MIRU types differed significantly in their drug resistance (Table 6). The largest proportion of MDR isolates was detected in types 100-32 and 4737-32 (both within B0/W148), 1065-32 (Central Asian/Russian), and 1071-32 (ancient sublineage). Central Asian/Russian strains included MIRU types heterogeneous in drug resistance. The largest type 94-32 included drug-susceptible and MDR strains in equal proportions (44.0% each). However, a closer look revealed that the 94-32 CAO subgroup included only drug-resistant (mostly MDR) isolates.

All isolates of type 9391-32 (modern sublineage but not Central Asian/Russian or B0/W148) were drug-susceptible. On the contrary, all six isolates of type 1071-32 (ancient sublineage) were MDR.

## 4. Discussion

The situation with TB in Russia is characterized by decreasing incidence (from 85.1/100,000 in 2008 to 41.2/100,000 in 2019), but increasing circulation of drug-resistant strains. The MDR-TB rate among newly-diagnosed patients in Russia increased from 13.0% in 2009 to 29.0% in 2018 [https://last.mednet.ru/images/materials/CMT/tuberkulez-2019.pdf, accessed on 1 December 2022]. Northwestern Russia has a somewhat better situation with TB compared to Siberia and Far East Russia (where TB/HIV coinfection presents a serious adverse factor). In Northwestern Russia, TB incidence decreased from 57.5/100,000 in 2010 to 25.2/100,000 in 2019 [28,29]. However, the primary MDR rate here is also high and knowledge of the high-resolution population structure is needed to timely identify the most hazardous *M. tuberculosis* variants, monitor their spread, and implement adequate TB control measures.

This study demonstrated that the *M. tuberculosis* Beijing genotype in Northwestern Russia is dominated by two large groups of the modern Beijing sublineage, Central Asian/Russian (61.5%) and B0/W148 (26.9%). Within the Central Asian/Russian clade, a Central Asia-successful CAO strain was detected at a minor prevalence rate (6.6% of all Beijing strains). The 24-MIRU-VNTR diversity of the B0/W148 and CAO strains was low (HGI = 0.41 and 0.46) suggesting their relatively more recent origin and more recent spread compared to Central Asian/Russian clade (HGI = 0.83).

All Beijing variants identified herein (except for CAO) are endemic Russian strains [7,12,18,21,22,23,30,31,32,33,34,35,36]. In this study, the vast majority of B0/W148 strains (88.3%) were assigned to four clusters of the CC2 clonal complex: 100-32 (86.8%), 4737-32, 1075-32, and 9378-32. Type 100-32 strains can be found in the former Soviet Union, Europe, and the USA (brought with FSU immigrants) [37,38,39,40,41], and in negligibly small prevalence rates in China, Korea, and Vietnam [2]. Russian B0/W148 strain likely originated in Siberia within the ancestral 100-32 subpopulation in the 1950s and further spread towards the European part of Russia in the 1960-1980s, followed by even wider dissemination by Russian immigrants in Europe and North America [37]. This conclusion was confirmed by a more recent WGS study [42].

The location of origin of the Russian Central Asian/Russian clade is presently unknown. The majority of these strains (79.0%) were assigned to 15 MIRU-VNTR types. Types 95-32 (6.8%; 12), 1065-32 (9.7%; 17), and the largest 94-32 (42.6%; 75) are widely distributed across the Russian Federation and post-Soviet countries, particularly dominating in Kazakhstan [34,35,36,39,40,41] although in the latter case, the contribution of the CAO strain is not known. For example, in Uzbekistan, the Central Asian/Russian clade is almost exclusively represented by the CAO strains [43]. The CAO strain is rarely found in Russia, e.g., 7% in Samara, Central Russia [44] and 6.4% in Omsk, Western Siberia [45]. Its presence in Omsk is not surprising since this Russian region borders northern Kazakhstan.

Regarding other minor variants, a drug-susceptible type 9391-32 (3% in this study) falls within type M41 in the 12-MIRU-VNTR database of Mokrousov et al. [46]. In that database, M41 is a minor variant that was detected >10 years ago in single isolates in the earlier studies in Northwestern Russia (Pskov, Kaliningrad, St. Petersburg) and was also described in Lithuania (4 drug-susceptible out of 157 Beijing isolates [2]). Finally, MDR/pre-XDR type 1071-32 belongs to the early ancient Beijing sublineage, recently discovered in Western Siberia at 10% and endemic to Russia; its secondary single isolates were described in parts of the Former Soviet Union and Eastern Europe [47].

The Central Asian/Russian clade (including CAO) had a significantly lower MDR rate (40.3%) than B0/W148 (85.7%). Comparative analysis of the phenotypic drug resistance of Beijing strains of the main MIRU-VNTR clusters showed a dominance of MDR strains in clusters 1071-32 (ancient sublineage, 100%), 4737-32 (B0/W148, 100%), 1065-32 (Central Asian/Russian, 88.2%) and 100-32 (B0/W148 (83.1%). Previously, MDR 4737-32 isolates were detected in Armenia, Lithuania, and some Western European countries, in particular, Portugal, with suggested FSU origin [2,48,49]. MDR type 1065-32 isolates were identified in Europe, with the largest proportion in Lithuania (32.7%; 17/52 Beijing strains) [2] and Estonia (31.0%; 9/29 Beijing strains) [50]. In our study, the largest proportions of MDR 1065-32 strains were found in the Pskov region bordering Estonia (35.3%; 6/17) but also in the inland Komi region (17.5%; 7/40). This reconfirms the Russian origin of this type that is geographically widespread in Russia (while time and location are unknown), and its subsequent penetration to Latvia and Estonia through human exchange with Russia.

While most of the studied provinces were borderline regions, the Vologda province takes a particular position (Figure 2). It is the inland Russian region and has a relatively calm epidemic situation for TB. In 2018, TB incidence here per 100,000 population was 15.8 compared to 28.6 in Northwestern Russia and 44.4 in Russia as a whole [28]. The Central Asian/Russian genotype was the most prevalent (66.7%) in Vologda where B0/W148 was at the lowest rate (11.8%). Indeed, this may account for a better situation with MDR-TB in this region. On the other hand, the higher prevalence rate of MDR-associated CAO in Vologda (13.7%) compared to other provinces of Northwestern Russia (2.9–8.7%) is noteworthy.

In this study, all CAO isolates (albeit low-prevalent, 3.8% of all *M. tuberculosis* collection) were drug-resistant and 58% of them were MDR. The CAO strain was shown to be MDR/pre-XDR, transmissible, and both endemic and epidemic in Central Asia [30,43], but its presence was rarely assessed in the previous Russian studies with few exceptions [18,31,43]. Unlike other Beijing variants described herein, Central Asian CAO is not an autochthonous Russian strain. Its wide dissemination in Central Asia was estimated to have started only in the 1990s after those countries gained their independence [43]. Penetration of the CAO cluster can be hypothetically attributed to the two different kinds of human migration. First, a significant migration of ethnic Russians from the former Soviet republics of Central Asia (Kazakhstan, Uzbekistan, and others) took place in the 1990s following the collapse of the Soviet Union [51,52,53]. More recently, already in the 21st century, there was a permanent and visible influx of work migrants from the same countries towards Russia. Detailed information on the volume and content of particular migration flows toward different Russian provinces is not available and any further considerations would be too speculative. Further surveillance is necessary to see if the CAO strains increase their circulation in Vologda and other targeted provinces.

## 5. Conclusions

The *M. tuberculosis* population in Northwestern Russia is dominated by the Beijing genotype (55.6%) and more than half of the Beijing strains were MDR. Two main Beijing groups B0/W148 and Central Asian/Russian differed significantly in MDR rate (85.7% vs. 40.3%, *p* < 0.0001). MDR-associated B0/W148 and CAO groups were dominated by single VNTR types (100-32 and 94-32) which implies the insufficient discriminatory capacity of 24-MIRU typing with regard to the epidemiologically significant Beijing types in Russia. In this view, the use of hypervariable loci may be recommended for further subtyping of these isolates. The largest VNTR types were 94-32, 1065-32, 95-32 (Central Asian/Russian), and 100-32 (B0/W148). Two-thirds of Beijing MDR strains were distributed among three MIRU-VNTR types: 100-32, 94-32, and 1065-32. The presence of MDR-associated, as yet minor genotypes (Central Asian CAO strain and Siberian 1071-32-cluster) in some of the studied provinces also requires attention. The observed difference between the studied provinces with regard to the regional population structures may be speculatively explained by (i) differences in local implementation of the TB control programs; (ii) unknown outbreaks; or (iii) demographic or migration-related factors.

The increasing rate of primary MDR-TB in Northwestern Russia can be explained by the active transmission of the Beijing genotype strains, primarily the B0/W148 cluster associated with MDR. This poses a serious problem since the treatment of MDR-TB patients requires significant economic costs.

The results of this study lay a foundation for epidemiological monitoring of the circulating *M. tuberculosis* strains and molecular epidemiology of tuberculosis in Northwestern Russia. Molecular epidemiology and permanent surveillance of the circulation of the epidemic, potentially emerging genotypes, or imported strains should become an integral part of the national TB control program.

## Figures and Tables

**Table 1:** *M. tuberculosis* Beijing genotype subgroups in the regions of Northwestern Russia.

Region, Number of Isolates	Number of Isolates (% of All Beijing)					All Beijing
	B0/W148	Central Asian/Russian (Other Than CAO)	Central Asian/Russian CAO	Other Modern Beijing	Ancient Beijing	
Vologda, *n* = 82	6 (11.8)	34 (66.7)	7 (13.7)	3 (5.9)	1 (2.0)	51 (62.2)
Kaliningrad, *n* = 73	17 (37.0)	17 (37.0)	4 (8.7)	6 (13.0)	2 (4.3)	46 (63.0)
Karelia *n* = 67	13 (36.1)	15 (41.7)	3 (8.3)	2 (5.6)	3 (8.3)	36 (53.7)
Komi, *n* = 130	26 (35.6)	43 (58.9)	2 (2.7)	1 (1.4)	1 (1.4)	73 (56.2)
Murmansk, *n* = 67	7 (20.0)	22 (62.9)	1 (2.9)	5 (14.3)	0	35 (52.2)
Pskov, *n* = 78	8 (17.8)	26 (57.8)	2 (4.4)	8 (17.8)	1 (2.2)	45 (57.7)

**Table 2:** Allele diversity of 24 MIRU-VNTR loci in *M. tuberculosis* strains Beijing Central Asian/Russian and B0/W148.

VNTR Locus	Locus Alias	Number of Alleles	Number of Repeats	HGI	Number of Alleles	Number of Repeats	HGI
		Central Asian/Russian (*n* = 176)			B0/W148 (*n* = 77)		
154	MIRU02	1	2	0	1	2	0
424	Mtub04	4	2–5	0.109	2	2.4	0.026
577	ETRC	3	2.4.5	0.067	1	4	0
580	MIRU04	1	2	0	1	2	0
802	MIRU40	3	2–4	0.078	2	3–4	0.026
960	MIRU10	2	2.3	0.011	1	3	0
1644	MIRU16	1	3	0	1	3	0
1955	Mtub21	5	1.3–6	0.120	2	4–5	0.051
2059	MIRU20	1	2	0	1	2	0
2165	ETRA	4	1–4	0.224	1	4	0
2347	Mtub29	3	2–4	0.077	1	4	0
2401	Mtub30	2	2.4	0.034	2	2.4	0.026
2461	ETRB	1	2	0	1	2	0
2531	MIRU23	1	5	0	1	5	0
2687	MIRU24	1	1	0	1	1	0
2996	MIRU26	4	4–6.9	0.161	3	4–6.7	0.077
3007	MIRU27	1	3	0	1	3	0
3171	Mtub34	2	2.3	0.011	1	3	0
3192	MIRU31	3	4–6	0.149	2	4–5	0.123
3690	Mtub39	2	3–4	0.011	3	2–4	0.052
4052	QUB26	5	5–9	0.382	3	2.3.7	0.052
4156	QUB4156	1	2	0	1	2	0
4348	MIRU39	2	2.3	0.011	1	3	0
2163b	QUB11b	5	2.5–8	0.170	2	5–6	0.076

**Table 3:** 24-MIRU-VNTR shared types (2 isolates and more) of main genotype groups.

Beijing Subgroups	24 Loci MIRU-VNTR Profile	MLVA MtbC 15-9	24-loci MIT	CC *	Number of Isolates
Central Asian/Russian *n* = 176	223325153533324682454433	9358-32	32	CC1	2
	223325153533424682444433	801-32	Orphan	CC1	2
	223325153533422682454433	9357-32	-	CC1	2
	223325153633422682454433	9356-32	71	CC1	2
	223325153533424582454433	407-32	-	CC4 *	2
	223325153534424582454433	770-32	-	CC4 *	2
	223325153433424682254433	?-32	-	-	2
	223325143533424682454433	1068-32	-	CC3 *	3
	223325153533424682464433	96-32	-	CC1	3
	223325153533424672452433	99-332	-	CC1	4
	223325163533424682454433	1076-32	Orphan	CC3 *	5
	223325153533424672454433	99-32	-	CC1	6
	223325153533324682454433	95-32	32	CC1	12
	223325153533424662454433	1065-32	28	CC1	17
	223325153533424682454433	94-32	25	CC1	75
B0/W148 *n* = 77	223325173533424672444433	9378-32	35	CC2	2
	223325163533424672454433	1075-32	-	CC2	2
	223325173433424672454433	4737-32	Orphan	CC2	5
	223325173533424672454433	100-32	27	CC2	59
Other modern Beijing, *n* = 25	244233352544425173353923	9380-32	-	CC4 *	2
	244233352644425173353623	1066-32	-	CC3 *	2
	244233352644425173353823	1048-32	-	CC3 *	2
	244234352644425173353823	9391-32	-	CC3 *	9
Ancient Beijing, *n* = 8	244231342644425173353923	1071-32	-	BL7	6

Note. Designations. The numerical profile of the strain shows the number of repeats in each of the 24 MIRU-VNTR loci in the following order: 12 loci MIRU, ETR-A, ETR-B, ETR-C, QUB-11b, QUB-26, QUB-4156, Mtub04, Mtub21, Mtub29, Mtub30, Mtub34, and Mtub39. CC is according to [2]. MIT refers to the SITVIT2 database. MLVA MtbC 15-9 refers to the code assigned to the MIRU-VNTRplus.org database. The MLVA MtbC15-9 type is defined by the combination of a type based on the 15 loci (MLVA MtbC15) of the discriminatory subset and a type based on the 9 auxiliary loci (MLVA MtbC9). * CC3 and CC4 [2] are polyphyletic groups not supported by robust WGS analysis.

**Table 4:** Regional VNTR-based diversity of *M. tuberculosis* Beijing genotype strains.

Region	Number of Isolates	Number of VNTR Types	Number of Clusters	Cluster Size	Number of Clustered Isolates (%)	CR	HGI	Shannon Index
Vologda	51	22	5	2–18	34 (66.7)	0.57	0.852	2.48
Kaliningrad	46	24	6	2–13	28 (60.9)	0.48	0.904	2.65
Karelia	36	18	3	3–9	21 (58.3)	0.50	0.881	2.55
Komi	73	23	8	2–21	58 (79.5)	0.68	0.854	2.28
Murmansk	35	13	4	2–15	26 (74.3)	0.63	0.790	2.04
Pskov	45	23	6	2–10	28 (62.2)	0.49	0.921	2.62

CR—clustering rate; HGI—Hunter Gaston Index.

**Table 5:** Drug resistance structure of the studied Beijing strains.

Drug Resistance	Number of Isolates and % of All of This Subtype					*p*
	B0/W148, *n* = 77	Central Asian/Russian (Other Than CAO), *n* = 157	Central Asian/Russian CAO, *n* = 19	Other Modern Beijing, *n* = 25	Ancient Beijing, *n* = 8	
Susceptible. *n* = 69	–	67 (42.6)	–	14 (56.0)	2 (25)	<0.001
Monoresistant/polyresistant *n* = 49	11 (14.3)	30 (19.1)	8 (42.1)	6 (24.0)	–	
MDR. *n* = 143	66 (85.7)	60 (38.2)	11 (57.9)	5 (20.0)	6 (75)	

Note. Polyresistant strain is resistant to at least two drugs but not MDR.

**Table 6:** Drug resistance of the main MIRU-VNTR clusters.

Beijing Clade	24-MIRU-VNTR Type	Number of Strains and % of All in This Type			*p*
		Susceptible	Monoresistant/Polyresistant	MDR	
B0/W148	100-32		10 (16.9)	49 (83.1)	*p* 100-32 vs. 1076-32 = 0.003 *p* 100-32 vs. 9391-32 < 0.001 *p* 100-32 vs. 94-32 < 0.001 *p* 100-32 vs. 95-32 = 0.008 *p* 100-32 vs. 99-32 < 0.001 *p* 1065-32 vs. 9391-32 < 0.001 *p* 1065-32 vs. 94-32 = 0.048 *p* 1065-32 vs. 99-32 = 0.010 *p* 1071-32 vs. 99-32 = 0.026 *p* 94-32 vs. 99-32 = 0.039
B0/W148	4737-32			5 (100)	
C.A.R.	94-32	33 (44.0)	9 (12.0)	33 (44.0)	
C.A.R.	1065-32		2 (11.8)	15 (88.2)	
C.A.R.	95-32	3 (25.0)	3 (25.0)	6 (50.0)	
C.A.R.	99-32	2 (33.3)	4 (66.7)		
C.A.R.	1076-32	1 (20.0)	3 (60.0)	1 (20.0)	
other	9391-32	9 (100)			
Ancient	1071-32			6 (100)	

C.A.R.—Central Asian Russian. *p* refers to the pairwise comparison of the genotypes. Only significant *p* values are shown (<0.05).

## Data Availability

All data of this study are presented in this article and Supplementary material.

## Supplementary Materials

The following supporting information can be downloaded at: https://www.mdpi.com/article/10.3390/microorganisms11020255/s1, Table S1: Information on all studied isolates of Mycobacterium tuberculosis Beijing genotype: origin, drug resistance, and 24-MIRU-VNTR loci profile.
